# Machine-learning based lipid mediator serum concentration patterns allow identification of multiple sclerosis patients with high accuracy

**DOI:** 10.1038/s41598-018-33077-8

**Published:** 2018-10-05

**Authors:** Jörn Lötsch, Susanne Schiffmann, Katja Schmitz, Robert Brunkhorst, Florian Lerch, Nerea Ferreiros, Sabine Wicker, Irmgard Tegeder, Gerd Geisslinger, Alfred Ultsch

**Affiliations:** 1 Institute of Clinical Pharmacology, Goethe-University, Theodor - Stern - Kai 7, 60590 Frankfurt am Main, Germany; 2 Fraunhofer Institute of Molecular Biology and Applied Ecology - Project Group Translational Medicine and Pharmacology (IME-TMP), Theodor - Stern - Kai 7, 60590 Frankfurt am Main, Germany; 3 Department of Neurology, Goethe-University Hospital, Theodor - Stern - Kai 7, 60590 Frankfurt am Main, Germany; 4 DataBionics Research Group, University of Marburg, Hans - Meerwein - Straße 22, 35032 Marburg, Germany; 5 Occupational Health Service, University Hospital Frankfurt, Theodor - Stern - Kai 7, 60590 Frankfurt am Main, Germany

## Abstract

Based on increasing evidence suggesting that MS pathology involves alterations in bioactive lipid metabolism, the present analysis was aimed at generating a complex serum lipid-biomarker. Using unsupervised machine-learning, implemented as emergent self-organizing maps of neuronal networks, swarm intelligence and Minimum Curvilinear Embedding, a cluster structure was found in the input data space comprising serum concentrations of d = 43 different lipid-markers of various classes. The structure coincided largely with the clinical diagnosis, indicating that the data provide a basis for the creation of a biomarker (classifier). This was subsequently assessed using supervised machine-learning, implemented as random forests and computed ABC analysis-based feature selection. Bayesian statistics-based biomarker creation was used to map the diagnostic classes of either MS patients (n = 102) or healthy subjects (n = 301). Eight lipid-markers passed the feature selection and comprised GluCerC16, LPA20:4, HETE15S, LacCerC24:1, C16Sphinganine, biopterin and the endocannabinoids PEA and OEA. A complex classifier or biomarker was developed that predicted MS at a sensitivity, specificity and accuracy of approximately 95% in training and test data sets, respectively. The present successful application of serum lipid marker concentrations to MS data is encouraging for further efforts to establish an MS biomarker based on serum lipidomics.

## Introduction

Multiple sclerosis (MS) is regarded as a chronic inflammatory, demyelinating and neurodegenerative autoimmune disease that affects the central nervous system^1^. In the most frequent relapsing-remitting form (RRMS), symptomatic periods alternate with longer periods of remission at disease onset but may eventually turn into secondary progressive disease^2^. Hence, the disease course is mostly characterized by a worsening of non-remitting clinical symptoms with each additional relapse^2^. The diagnosis, currently based on clinical parameters, the number, size and location of lesions detected by MRI and spinal fluid diagnostics, is often delayed due to heterogeneous symptoms and long recovery phases at the beginning of the disease^2^, thus preventing timely therapy initiation^3^, and other neurologic diseases may mimic the symptoms in early phases^4–6^. The search for biomarkers to improve the diagnosis of MS is an active research topic^7^. Approaches include positron emission tomography addressing neuro-inflammation and astrocyte markers^8^, genetic, immune-inflammatory, and oxidative stress markers^9^, Vitamin D binding protein isoforms and apolipoprotein E in cerebrospinal fluid^10^, and plasma micro RNAs^11,12^. Further blood-based biomarkers utilize metabolomic^13^ and proteomic markers^14^ or serum profiles of cytokines, chemokines and pro-apoptotic molecules^15^.

Lipid metabolism has been suggested, among others^1^, to be a major pathophysiological mechanism of multiple sclerosis (MS)^16^, even that MS is in fact a disease due to disturbed lipid metabolism^17^. Among lipids, cholesterol and cholesterol turnover products have been associated with MS^18^, whereas omega-3 lipids were protective by preserving the blood brain barrier^19^. Recent investigations point at several further classes of lipids that are regulated in MS. Currently, a scientific focus centers on prostaglandins, hydroxyeicosatetraenoic acids^20^, ceramides and lysophosphatidic acids^21–23^. The successful therapy of human MS with fingolimod, which antagonizes functions of sphingosine-1-phosphate (S1P) highlights the pathophysiologic relevance of bioactive lipids. In addition, recent research addressing ceramides in MS show that these lipids modify the course of experimental MS models^22,24^. The benefits of cannabinoids for symptomatic control of MS-associated pain and muscle spasms^25–27^ and experimentally proven anti-neuro-inflammatory effects of cannabinoids^28,29^ further suggest a contribution of bioactive lipids to symptom control, resolution of inflammation and possibly remyelination^17^.

Considering the complexity of the lipidome, we searched for a lipidomics based biomarker for MS diagnosis and assessment of therapeutic efficacy^18,30^. This is in line with the evidence that ceramides, lysophosphatidic acids (LPA)^21,22^, endocannabinoids^31^ or eicosanoids^20^ are dysregulated in MS patients. Interference with the metabolism or receptor action of these lipids modifies the course of the disease in experimental autoimmune encephalomyelitis (EAE) models of multiple sclerosis in rodents^25,32–35^ and fingolimod shows that S1P is a key regulator of MS in humans. To analyze the potential utility of a complex lipid based MS diagnostic approach, we have developed sensitive assays for d = 43 different bioactive lipid serum markers of various classes (ceramides, sphingolipids, lysophosphatidic acids (LPAs), endocannabinoids, pterins, prostaglandins, dihydroxyeisocatrienoic acids (DHETs), and hydroxyeicosatetraenoic acids (HETEs). As most single markers are also regulated in cancer, atherosclerosis or ischemia, a complex biomarker was targeted. Using machine learning techniques^36^, the present investigation aimed at the following. (i) To establish whether the serum concentration patterns of d = 43 lipid markers are suitable for the identification of multiple sclerosis patients. (ii) To identify the combination of lipid markers (features^37^) in a reduced set that is accessible to biomedical mechanistic interpretation and not unnecessarily burdening to the laboratory analytical resources which provides a classifier or biomarker to discriminate between an MS patient or a healthy subject with high accuracy.

## Methods

### Subjects and study design

The study followed the Declaration of Helsinki and was approved by the Ethics Committee of the Medical Faculty of the Goethe – University Frankfurt am Main, Germany. Informed written consent was obtained from all subjects. Employing a parallel group design, patients with multiple sclerosis (n = 102, aged 18.2–62.8 years, 31 men) and healthy controls (n = 301, aged 18–53.2 years, 118 men) were consecutively recruited from outpatients and inpatients of the Department of Neurology (patients) and from students and staff members of the hospital (controls) who routinely reported to the institutional occupational health service. Patients and healthy subjects showed a similar sex distribution (χ^2^-test^38^: χ^2^ = 2.1738, degrees of freedom, df = 1, p = 0.1404) but different ages (Wilcoxon signed rank test^39^: W = 25,834, p < 2.2 · 10^−16^), which was taken into consideration during data preprocessing (see respective section below). Data and blood collection from MS patients was part of the local bio-banking project (Neurological Department of the Goethe University, Frankfurt). Inclusion criteria were age ≥18 years, for patients, a clinically verified diagnosis of multiple sclerosis based on McDonald criteria and for controls, no current medical condition queried by medical interview, and no drug intake for at least one week except contraceptives, vitamins and L-thyroxin. Demographic data including time since diagnosis, Expanded Disability Status Scale (EDSS)^40^ and current disease modifying medication are summarized in Table 1.

**Table 1:** Demographic parameters of the MS patients, disease characteristics and medications.

	MS 1^st^ course						RRMS no relapse						RRMS acute relapse						SPMS or PPMS					
	Mean	SD	n	Mean	SD	n	Mean	SD	n	Mean	SD	n	Mean	SD	n	Mean	SD	n	Mean	SD	n	Mean	SD	n
Sex	m			f			m			f			m			f			m			f		
Age	38.2	7.5	8	31.6	9.1	12	35.5	9.4	16	34.3	8.9	39	33.8	7.0	4	37.2	10.9	18	43.6	18.1	3	49.2	11.0	2
Disease years	0.03	0.04		0.9	2.9		7.5	6.1		7.4	5.9		2.1	1.8		6.7	5.3		5.7	4.7		1.0	1.3	
EDSS	0.6	1.0		0.8	1.1		3.1	1.7		2.3	1.6		1.63	1.2		2.0	1.9		4.0	2.8		5.0		
Medication																								
None			6			11			3			6			1			10			1			1
β-Interferon			0			0			6			11			2			5			0			1
Fingolimod			0			0			3			5			0			1			1			0
Natalizumab			0			0			4			15			0			0			0			0
Other			2			1			0			2			1			2			1			0

EDSS: Expanded Disability Status Scale, RRMS: Relapsing Remitting MS, PPMS: Primary Progressive MS, SPMS: Secondary Progressive MS.

### Lipid mediator serum concentration analysis

**Figure 1:**
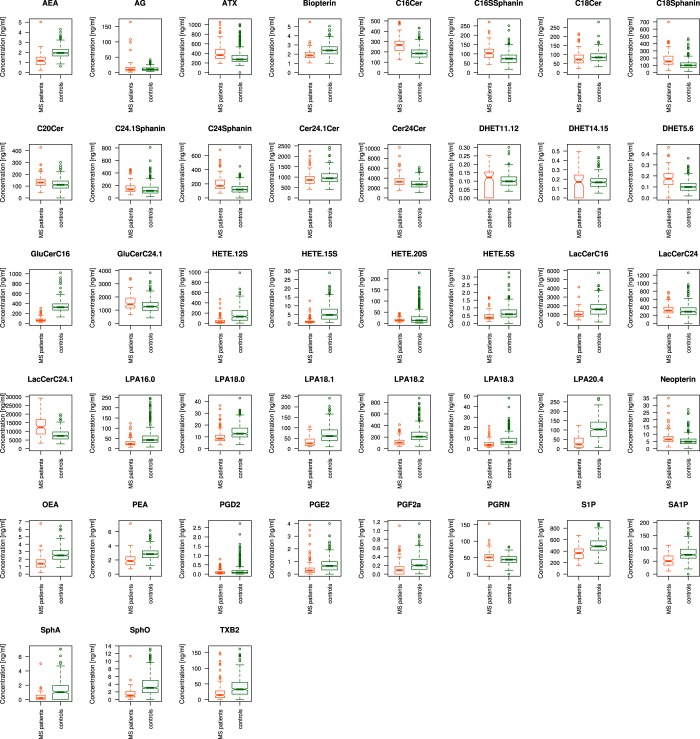
Serum concentrations of d = 43 lipid mediators (raw data). The data are shown in alphabetical order of marker names and for each marker, separately for group membership to the multiple sclerosis patients (left boxes, red) or the healthy subjects (right boxes, green). The widths of the boxes are proportional to the respective numbers of subjects per group. The quartiles and medians (solid horizontal line within the box) are used to construct a “box and whisker” plot. The whiskers add 1.5 times the interquartile range (IQR) to the 75^th^ percentile or subtract 1.5 times the IQR from the 25th percentile and are expected to include 99.3% of the data if normally distributed. The notches indicate the confidence interval around the median based on *median* ± 1.57 · *IQR*/*n*^0.5^. The figure has been created using the R software package (version 3.4.2 for Linux; http://CRAN.R-project.org/)^43^.

From each subject, a venous blood sample (9 ml) was collected into a serum tube and centrifuged at 3,000 rpm for 10 min. Serum was separated and frozen at −80 °C until assay. A total of d = 43 different lipid mediators (Fig. 1) was analyzed from the serum samples. The selection included ceramides (Cer16:0, Cer18:0, Cer18:1, Cer20:0, Cer24:0, Cer24:1, GluCerC16:0, GluCerC24:1, LacCerC16:0, LacCerC24:0, LacCerC24:0), lyosophosphatidic acids (LPA16:0, LPA18:0, LPA18:1, LPA18:2, LPA18:3, LPA20:4)), sphingolipids (sphinganine, sphingosine, S1P, SA1P C16Sphinganine, C18Sphinganine, C24Sphinganine, C24:1Sphinganine), prostaglandins (PGD2, PGF1α, PGE2, TXB2), dihydroxyeicosatrienoic acids (DHET5.6, DHET11.12, DHET14.15), hydroxyeicosatetraenoic acids (HETE 5 S, HETE_12S, HETE_15S, HETE_20S), endocannabinoids (AEA, OEA, PEA, 2-AG) and pterins (biopterin, neopterin). Pterins were included because tetrahydrobiopterin regulates the metabolism of multiple bioactive lipids as a coenzyme of alkylglycerolmono-oxygenase (AGMO)^41^.

Serum concentration analyses were performed using liquid chromatography-electrospray ionization-tandem mass spectrometry (LC-ESI-MS/MS) as described previously^41,42^. In brief, eicosanoids (DHET11.12, DHET14.15, DHET5.6, HETE12S, HETE15S, HETE20S, HETE5S, PGD2, PGE2, PGF2α, TXB2; PGD2 = prostaglandin D2, PGE2 = prostaglandin E2, PGF2α = prostaglandin F2a, TXB2 = thromboxane, DHET = dihydroxyeicosatrienoic acid, HETE = hydroxyeicosatetraenoic acid) were assayed in two different LC-MS/MS runs. Prostanoids were separated using a Synergi Hydro column (150 × 2 mm, 4 µm, Phenomenex, Aschaffenburg, Germany) and the analysis of HETE/DHET was done using a Gemini NX column (150 × 2 mm, 5 µm, Phenomenex). In both cases, quantification was performed using a triple quadrupole mass spectrometer QTRAP 5500 (Sciex, Darmstadt, Germany) equipped with a Turbo-V-source operating in negative ESI mode. Ceramides (C16Cer, C18Cer, C20Cer, C24Cer, C24:1Cer, GluCerC16, GluCerC24:1, LacCerC16, LacCerC24, LacCerC24:1; Cer = ceramide, GluCer = glucosylceramide, LacCer = lactosylceramide) were analyzed using a Luna C18 column (150 × 2 mm ID, 5 µm particle size, Phenomenex) coupled to an API 4000 mass spectrometer equipped with an APCI (Atmospheric Pressure Chemical Ionization) ion source operating in positive mode (Sciex). The analysis of lysophosphatidic acids (LPA16:0, LPA18:0, LPA18:1, LPA18:2, LPA18:3, LPA20:4) was performed using a Mercury Luna C18 column (20 × 2 mm, 3 µm, Phenomenex) coupled to a triple quadrupole mass spectrometer (QTRAP 5500) operating in negative ESI mode. In all cases, the analytes were extracted using liquid-liquid-extraction prior to LC-MS/MS-analysis. Sample volumes were 200 µl each for prostanoids and DHET/HETE, 20 µl for ceramides and 50 µl for LPA and endocannabinoids. For all analytes, the concentrations of the calibration standards, quality controls and samples were evaluated by Analyst software 1.6 and MultiQuant Software 3.0 (Sciex) using the internal standard method (isotope-dilution mass spectrometry). Calibration curves were calculated by linear regression with 1/x weighting for ceramides and LPA and by quadratic regression with 1/x^2^ weighting for eicosanoids.

### Data analysis

**Figure 2:**
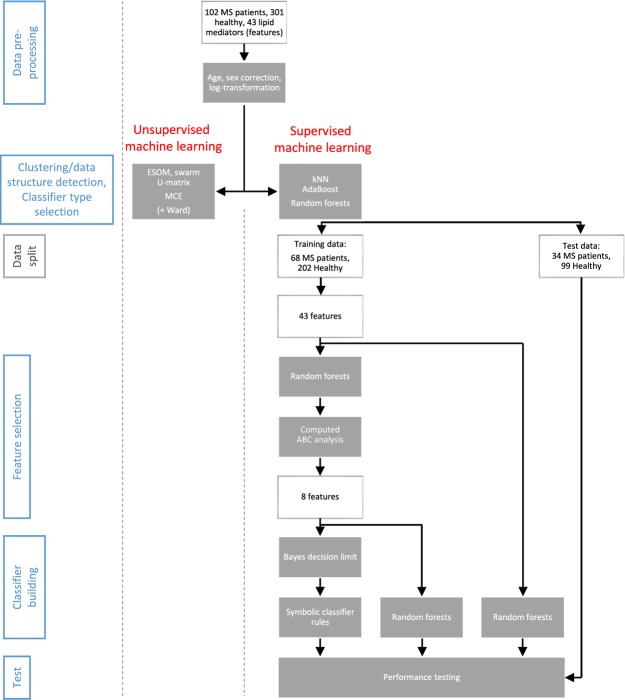
Flow chart of the data analysis. The figure provides an overview on the applied machine-learning approach in three main steps (indicated at the left side: data preprocessing, feature selection and classifier building including testing). The white frames show the variable flow, along with group size information. The grey frames depict the bioinformatics operations applied on the variables. During feature selection, the number of variables qualifying as components of a diagnostic tool respectively classifier was stepwise reduced, forwarding to the next analytical step only those features that had passed the criteria of the actual selection procedure.

Data were analyzed using the R software package (version 3.4.2 for Linux; http://CRAN.R-project.org/^43^) on an Intel Xeon® computer running on Ubuntu Linux 16.04.3 64-bit. The acquired parameters, subsequently called “features”, included d = 43 lipid mediators assayed from the participants’ venous blood serum (Fig. 1). The analysis was performed in five main steps (Fig. 2) comprising (i) data preprocessing, (ii) identification of a subject’s cluster structure for the lipid markers that coincided with the clinical diagnosis, (iii) selection of a suitable machine-learning method for biomarker creation, (iv) classifier (biomarker) building including feature selection and performance testing and (v) biomedical interpretation of the identified subset of lipid markers sufficient to diagnose MS.

#### Data preprocessing

A single outlier in the DHET5.6 serum concentrations was eliminated on the basis of a significant Grubbs test^44^ (G = 19.624, U = 0.03966, p < 2.2 · 10^−16^). Subsequently, data were preprocessed to correct for age and sex effects (significant differences in correlation analyses^45^ or Wilcoxon signed rank tests^39^ in several parameters, details not shown) based on linear regression or median differences, respectively. Following this correction, the statistically significant effects of age and sex were eliminated (age: Spearman’s ρ^45^ non-significant in all markers; sex: Wilcoxon signed rank tests^39^ non-significant in all markers. Quantile-quantile plots suggested a zero invariant log-transformation to *LogConcentration* = *ln* (*Concentration* + 1), which is in line with general advices for transformations of blood-concentration data^46^.

#### Identification of data cluster structures

The idea behind this analytical step was that if these clusters of subjects, obtained from the lipid marker concentration patterns, were to agree with the clinical diagnosis, then the data set was likely to be relevant to the clinical diagnosis of interest and thus, to provide a suitable basis for the creation of a biomarker (classifier). As common clustering algorithms such as k-means, Ward, complete- and average linkage^47^ are prone to detect false structures in the data^48^, unsupervised machine learning was used, implemented as a topology-preserving emergent self-organizing feature map (ESOM) (Kohonen SOM^49,50^) combined with the U-matrix^51^. We have shown recently that this method outperforms classical clustering algorithms in detecting the correct structures in artificial data sets and, in contrast to others, does not detect false structures in structure-less artificial or biomedical data sets^48^. As supporting methods, a swarm based projection^52^ and Minimum Curvilinear Embedding^53^ were used. Furthermore, the classical Ward clustering algorithm^54^ was applied to reassess subject clusters.

In unsupervised machine-learning, the goal is to find “interesting” structures in an unlabeled data set. When the identified distance and density based data structures coincided with the known diagnostic groups, i.e., in MS or healthy individuals, the d = 43 lipid markers were regarded as providing information relevant for the grouping of the cohorts into either patients or controls. This indicated whether the lipid marker concentrations were suitable for the separation of the two groups. The positive expectation was based on a recent analysis of sections of the present data set^55^. This earlier analysis, however, was aimed at analyzing separately the particular classes of lipids (eicosanoids, ceramides and lysophosphatidic acids) for their association with the MS diagnostic status rather than at biomarker creation. The data space $D=\{xi\in X\subset {R}^{d},\,i=1,\ldots n\}$ comprising the concentrations of d = 43 lipid markers (zero invariant log-transformed and subsequently normalized to percentages) acquired from n = 403 subjects, was explored for structures that possibly overlapped with the known data labeling or the group structure of MS patients or controls.

ESOM^49^ are based on a topology-preserving projection of high-dimensional data points $xi\in X\subset {R}^{d}$ onto a two dimensional self-organizing network consisting of a grid of neurons. The neural network consisted of a two-dimensional toroid grid^51^ of so-called neurons with 50 rows and 80 columns (n = 4,000 units, for SOM size determination, see^48^). Each neuron holds, in addition to a position vector on the two-dimensional grid, a further vector carrying “weights” of the same dimensions as the input dimensions. The weights were initially drawn randomly from the sets of data variables and subsequently, adapted to the data during the learning phase with 20 epochs. Following training of the neural network, an ESOM was obtained that represented the subjects on a two-dimensional toroid map as the localizations of their respective “best matching units” (BMU). These were neurons on the grid, which after ESOM learning, carried the vector that was most similar to a subject’s data vector. These calculations were performed using our R package “Umatrix” (https://cran.r-project.org/package=Umatrix)^56^.

An alternative data projection was obtained using a swarm of intelligent agents called DataBots, i.e., self-organizing artificial “life forms” that carry vectors of the biological processes associated with the drugs via their genetic targets. The data space was explored for distance-based structures. A parameter-free focusing projection method of a polar swarm, *Pswarm*, was used that exploits concepts of self-organization and swarm intelligence. Following successful swarm learning, DataBots carrying items with similar features were placed in groups on the projection grid. The identification of emergent structures in the learned structure was further enhanced. These calculations were performed using the R library “DatabionicSwarm” (M. Thrun, https://cran.r-project.org/package=DatabionicSwarm)^57^.

At the top of the grids on which the data had been projected using either ESOM or swarm based methods, the distances between data points were calculated using the so-called U-matrix^47,58^. Every value (height) in the U-matrix depicts the average high-dimensional distance of a prototype in relation to all immediate neighboring prototypes with regard to grid position. The corresponding visualization technique is a topographical map with hypsometric colors^59^ facilitating the recognition of distance and density based structures. Large “heights” in brown and white colors represent large distances between data.

To further explore the structure of the input data space, unsupervised machine learning was additionally implemented as Minimum Curvilinear Embedding^53^, which is a parameter-free nonlinear data projection method^60^ that uses a so called geodesic distance. It assumes that the high dimensional data reside basically on lower dimensional sub-manifolds, which can be effectively represented by a neighborhood graph. Within this graph, the data distances are defined as geodesic distances. The most prominent of these projection methods is the Isomap algorithm^61^. MCE uses the minimal spanning tree as such a graph structure. In the present analysis, a minimum curvilinearity kernel was built on the Euclidean and correlation distance matrices provided by the input features, either in the non-preprocessed or in the age and sex corrected data versions, using both centered and non-centered versions of the implementation. These calculations were done using a script downloaded from https://sites.google.com/site/carlovittoriocannistraci/5-datasets-and-matlab-code/minimum-curvilinearity-ii-april-2012 and the library “igraph” (Csárdi G. https://cran.r-project.org/web/packages/igraph/index.html)^62^. Finally, a further analysis of group structure in the data consisted of Ward clustering, which was implemented using the R library “cluster” (M. Maechler, https://cran.r-project.org/package=cluster)^63^.

#### Selection of machine-learning methods for classifier creation

For classifier or biomarker creation, supervised machine learning was employed. In the supervised machine learning^36^ approach, the goal is to learn mapping from inputs *x* to output *y*, given a labeled set of input-output pairs $D=\{({x}_{i},{y}_{i})|{x}_{i}\in {\rm{X}},{y}_{i}\in {\rm{Y}},i=1\ldots n\}\,$, composed of pairs of Boolean values *y*_*i*_ ∈ *Y* = *B* comprising the diagnoses of either “multiple sclerosis” (1) or “healthy” (0), and of values ${x}_{i}\in X\subset { {\mathcal R} }^{d}$ comprising the *d* features, respectively serum concentrations of lipid markers, possibly predicting these diagnoses. Three different supervised machine-learning methods were explored with respect to the classification performance they provided in the present data set, comprising (i) k-nearest neighbors^64^, (ii) adaptive boosting^65^ and (iii) random forests^66^.

The k-nearest neighbor (kNN) classification^67^ is a non-parametric method during which the entire labeled training dataset is stored, while a test case is placed in the feature space in the vicinity of the test cases at the smallest high dimensional distance. The data space consisted of the zero invariant log-transformed lipid marker serum concentrations compatible with the Euclidean distance, subsequently normalized to percentages, and the diagnosis classes. The test case is given a class label according to the majority vote of the class labels of the *k* training cases in its vicinity. In the present implementation, the size of *k* was set at a value of 5, which is the default of the R package “KernelKnn” (Mouselimis L, https://cran.r-project.org/package=KernelKnn) used for these calculations.

Boosting^65^ employs a weak learning algorithm. Initially, each of n data points is associated the same weight *w*_*i*_ = *1/n*. A learner is implemented as a small classification and regression tree^68^, which provides a simple form of classification rules, using the Gini impurity (for details, see https://en.wikipedia.org/wiki/Decision_tree_learning #Gini_impurity) to find optimal (local) dichotomic decisions. The data space consisted of the zero-invariant log transformed lipid marker serum concentrations and the diagnosis classes. The final model combined all models using a weighted sum of the outputs that reflect the accuracy of all the constituent models. The number of iterations was heuristically based on the classification accuracy, which indicated no improvement beyond 500 runs. These calculations were done using the R package “ada” (http://cran.r-project.org/package=ada^69^ with the partitioning and classification package “rpart” https://cran.r-project.org/package=rpart).

Random forests create sets of different, uncorrelated and often very simple decision trees^66^ with conditions of features shown as vertices and classes as leaves. The splits of the features are random and the classifier relates to the majority vote for class membership provided by a large number of decision trees. The data space consisted of the zero-invariant log transformed lipid marker serum concentrations and the diagnosis classes. In the present analysis, 1,000 decision trees were built containing *sqrt(d)* features or nucleotide positions as the standard setting implemented in the R library “randomForest” (https://cran.r-project.org/package=randomForest)^70^. The number of trees was heuristically based on visual analysis of the relationship between the number of decision trees and the classification accuracy, which indicated that beyond 100 trees, the classification balanced accuracy remained stable and a larger number merely consumed available computation time.

#### Classifier creation

As random forests provided the best classifier during the machine-leaning methods comparison, the biomarker was created using a combination of a random forests approach combined with Bayesian statistics as described in the following. The classifier is firstly trained in a labeled data set and subsequently applied to novel data where its diagnostic performance is assessed. To this end, the original data set was randomly split into a 2/3 sized training data set and a 1/3 sized test data set that both contained the two study groups in size proportional counts.

Feature selection: To obtain a classifier accessible for functional interpretation, and to avoid unnecessary laboratory analytics in the future, a feature selection step was included in the analysis with the aim to identify, among the d = 43 lipid markers, a subset on which a sensitive diagnosis could be based. Feature selection for classifier development was implemented as random forest analysis^66^ followed by computed ABC analysis^71^. Random forests create sets of different, uncorrelated decision trees^66^ with conditions of variables (features) as vertices and classes as leaves. Each tree in the random forest votes for a class and the final classification assigned to a data point follows the majority of these class votes. In the present analysis, 500 decision trees containing up to six randomly drawn features were calculated from data with equal group sizes randomly drawn from the training data set. The number of trees was heuristically based on visual analysis of the relationship between the number of decision trees and the classification accuracy. This indicated no improvement beyond 100 trees, so 500 trees were considered to provide robust results. To establish that these heuristics did not affect the results of classifier creation, different numbers of trees such as 100 or 1,000 were also tested, which always led to the selection of the same lipid markers as members of the final biomarker.

From these trials, features or lipid markers were included into the final classifier based on the mean decrease in classification accuracy, when the respective feature was excluded from random forest building. The extent of this decrease indicated the importance of the particular feature. This procedure was repeated on 1,000 different training and test data sets, randomly drawn from the original training data set. Following the concept of a nested cross-validation analysis^72^, the random forest analysis was performed using randomly drawn sub-samples from the actual training sample and the forest was applied to the actual test sample. These calculations were done using the R library “randomForest” (https://cran.r-project.org/package=randomForest)^70^.

After each random forest analysis, the values for the mean decrease in tree classification accuracy, when the feature was excluded from random forest analysis, were subsequently submitted to computed ABC analysis^73^. This is a categorization technique for the selection of the most important subset among a larger set of items. It was chosen since it fitted the basic requirements of feature selection using filtering techniques^74^. Thus, it easily scales to very high-dimensional datasets, is computationally simple and fast, and independent of the classification algorithm. Computed ABC analysis aims to divide a set of data into three disjointed subsets called “A”, “B” and “C”^75^. Subset “A” contains the most profitable features^76,77^ and was therefore, chosen for classifier establishment. For each of the 1,000 runs, the size and members of ABC set “A” were retained. The final size of the feature set was equal to the most frequent size of set “A” in the 1,000 runs, and the final members of the feature set were chosen in decreasing order of their appearance in ABC set “A” among the 1,000 runs. These calculations were done using our R package “ABCanalysis” (http://cran.r-project.org/package=ABCanalysis)^73^.

Before creating the final classifier, each of the selected features was judged by a topical expert who verified that no major evidence exists in the published medical literature that the feature is unsuitable to be included in a diagnostic tool for multiple sclerosis. An exclusion criterion, for instance, would be that the parameter is known to be regulated or used as a marker for non-MS CNS diseases, such as pathogen-caused or autoimmune-mediated inflammatory CNS diseases, neuroborreliosis, brain tumor, spinal ischemia, sarcoidosis, vasculitis, acute disseminated encephalopathy or leukodystrophy.

Classifier building: Having passed the above feature selection, the lipid markers were subsequently used to generate the classifier. To this end, for each feature f, a classification rule ${\rm{\psi }}(f)\to Y$ was used to assign a class label to the data on the basis of the observation of its feature vector. This was done by partitioning the feature space into two decision regions *F*_*1*_, *F*_2_ (yes or no for the diagnosis “multiple sclerosis”, respectively). For each feature *f* the probability density function (PDF) of the zero invariant log-transformed lipid mediator concentrations was modeled by optimizing a Gaussian mixture model (GMM) to the distribution. The GMM was given by

1 $$p({x}_{f})={\sum }_{i=0}^{M}{p}_{i}({x}_{f})=\,{\sum }_{i=0}^{M}{w}_{i}N({x}_{f}|Mea{n}_{i},S{D}_{i})={\sum }_{i=1}^{M}{w}_{i}\cdot \frac{1}{\sqrt{2\cdot \pi \cdot S{D}_{i}}}\cdot {e}^{-\frac{{({x}_{f}-Mea{n}_{i})}^{2}}{2\cdot S{D}_{i}^{2}}}$$

where *N(x*_*f*_*|Mean*_*i*_*, SD*_*i*_) denotes Gaussian probability densities (component, mode) with means, *Means*_*i*_ and standard deviations, *SD*_*i*_. The *w*_*i*_ are the mixture weights indicating the relative contribution of each component Gaussian to the overall distribution, which add up to a value of 1. *M* denotes the number of components in the mixture. GMM fitting and optimization was performed using the R package “AdaptGauss” (https://cran.r-project.org/package=AdaptGauss)^78^.

The Bayesian decision rule ${\rm{\psi }}(f)=1,\,if\,{p}_{i}(f) > {p}_{j}(f)\,and\,mean\,(f|\mathrm{multiple}\,{\rm{sclerosis}}) > \,mean\,(f|\mathrm{healthy})$, *else* 0 was used to assign a decision score to each feature f. This identified specific “yes/no” thresholds for each of the features. The final classifier was created by identifying a classification rule ${{\rm{\psi }}}_{d}({\rm{t}}):{ {\mathcal R} }^{d}\to Y,{{\rm{\psi }}}_{d}({\rm{t}})=SF > t$ that assigned a class label to the data based on the sum of decision scores SF of the features, where t is a threshold on this sum. All possible values of the threshold t from 1 to |{ABC set A}| were iteratively assessed with respect to the product of sensitivity and specificity. This identified an optimum threshold for patient classification. In this classifier, the relevant lipid mediator concentrations were represented by rules that are comprehensible for biomedical experts, which according to artificial intelligence concepts defines a symbolic classifier^79^.

An alternative sub-symbolic classifier was generated by means of random forest machine learning using the same |{ABC set A}| lipid markers as used in the symbolic classifier. In this classifier, the mediator concentrations are represented by the many trees of the forest. This eluded direct interpretation by biomedical experts since the classification can be obtained, but the details of the lipid mediators triggering the decision are hidden. Finally, a further random forest based classifier was created using the complete set of d = 43 lipid markers, with a complete nested cross validated approach and additional randomization of the number of features included in each tree and the number of trees built in each run. This procedure was implemented in 1,000 runs on resampled data.

Classifier performance testing: The performances of all classifiers were assessed using the test data set drawn up at the start of the data analysis and comprised the calculation of standard measures of test performance (sensitivity, specificity, balanced accuracy). In addition, the area under the ROC curve (AUC-ROC) and the area under the precision-recall curve (AUPRC) were calculated using the R libraries “pROC” (Robin X, https://cran.r-project.org/package=pROC)^80^ and “MLmetrics” (Yan Y, https://cran.r-project.org/package=MLmetrics), respectively. The 95% confidence intervals of the performance test parameters were obtained as the 2.5^th^ and 97.5^th^ percentiles of the results of 1,000 runs on Bootstrap resampled data.

## Results

Serum concentrations of lipid mediators were available from n = 102 patients with multiple sclerosis and n = 301 healthy subjects (Fig. 1). From these data, we first established a structure that coincided with the clinical picture of MS versus healthy group structure of the study cohort. This provided a basis to consider the lipid markers as suitable, in principle, to build a diagnostic biomarker for MS. Such classifiers were created in the second step of the analysis.

### Lipid mediator serum concentration patterns based subject clustering

**Figure 3:**
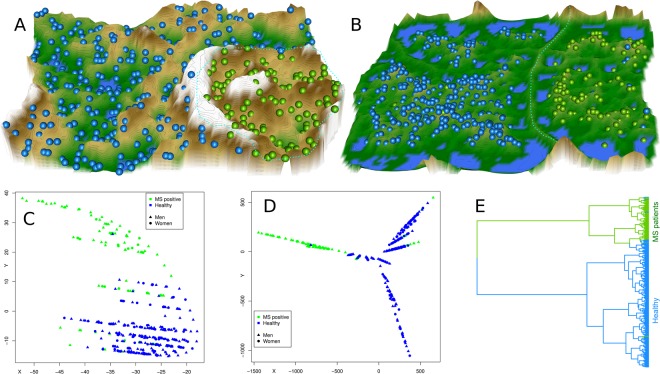
Clustering of subjects based on lipid marker serum concentrations, obtained using unsupervised machine learning (**A**–**D**) or classical Ward clustering (**E**). (**A**) U-matrix visualization of distance based structures of the serum concentration of d = 43 markers observed in n = 102 multiple sclerosis patients (green dots) and n = 301 healthy subjects (blue dots). The figure has been obtained using a projection of the data points onto a toroid grid of 4,000 neurons where opposite edges are connected. The dots represent the so-called “best matching units” (BMU), i.e., neurons on the grid that after ESOM learning carried the vector that was most similar to a subjects’ data vector. The U-matrix visualization was colored as a top view of a topographic map with brown (up to snow-covered) heights and green valleys with blue lakes. Watersheds indicate borderlines between different clusters (marked with a light blue dotted line). Superimposing the clinical diagnosis almost completely coincided with the cluster separation (accuracy 98%). (**B**) U-matrix visualization of the data structure found via a projection onto a toroid neuronal grid using a parameter-free polar swarm, *Pswarm* consisting of so-called DataBots, which self-organizing artificial “life forms” that carry vectors of the biological lipid marker concentrations. Superimposing the clinical diagnosis almost completely coincided with the cluster separation. (**C**,**D**) Minimum Curvilinear Embedding^53^ using the Euclidean distance matrix of the preprocessed data for kernel building and both, the non-centered (**C**) and the centered (**D**) versions of the method. Agreement with the clinical diagnosis can be concluded from the color-coded separation of the data. (**E**) Cluster structure found using classical Ward clustering. Agreement with the clinical diagnosis was slightly lower than with the machine-learned methods. The figure has been created using the R software package (version 3.4.2 for Linux; http://CRAN.R-project.org/)^43^. Specifically, for panel A our R package “Umatrix” (https://cran.r-project.org/package=Umatrix)^56^ was used, for panel B the library “DatabionicSwarm” (M. Thrun, https://cran.r-project.org/package=DatabionicSwarm)^52^, for panels C and D a script downloaded from https://sites.google.com/site/carlovittoriocannistraci/5-datasets-and-matlab-code/minimum-curvilinearity-ii-april-2012 and the library “igraph” (Csárdi G. https://cran.r-project.org/web/packages/igraph/index.html)^62^ was used, and for panel E the R library “ape”(https://cran.r-project.org/package=ape)^105^ was used.

Serum concentrations of d = 43 lipid markers were available from 102 patients with multiple sclerosis and 301 healthy subjects Fig. 1). Unsupervised machine learning, applied to identify structures in the data space $D=\{{x}_{i},\,i=1,\,\ldots \,,403\}\subset {{\mathbb{R}}}^{d}$, provided an emergent self-organizing feature map (ESOM), in which large U-heights indicated a large gap in the data space, whereas low U-heights indicated that the points are close to each other in the data space, indicating structure in the data set (Fig. 3A). On the topographic map of the U-matrix, valleys, ridges and basins enhance the visibility of the structure of clusters. From this structure, a “mountain range” separated two regions, which indicates the emergence of two main clusters in the data. Superimposing onto the cluster structure the class labeling into MS patients or control indicated an almost perfect separation of the diagnostic groups by the cluster structure. This was reflected in a 98% balanced accuracy of diagnosis assignment by the obtained cluster assignment of the subjects. The alternative approaches to subject subgroup detection, implemented as swarm based projection (Fig. 3B), Minimum Curvilinear Embedding (Fig. 3C,D), and as classical Ward clustering (Fig. 3E), also supported a data structure in the input space that coincided with the diagnostic groups. For the results of MCE, an apparent spilt of the healthy subjects into two subgroups (Fig. 3C,D) did not show any tendency toward a dominance of either sex, which ruled out a possible effect of contraceptive usage as an obvious explanation. From these analyses, it was concluded that the set of lipid markers included information suitable to separate MS from controls, which had been the aim of this data analysis step.

### Supervised machine-learning derived MS biomarker

The final comparative assessment of classifier performance provided by k-nearest neighbors, adaptive boosting and random forests, using the complete feature sets indicated a slight advantage of the random forests based classifier (Table 2). Therefore, this method was chosen as the basis for feature selection and biomarker creation.

**Table 2:** Test performance measures for the correct prediction of the diagnosis “multiple sclerosis” provided by different types of classifiers obtained using k-nearest neighbors (kNN), adaptive boosting or random forests. The first three implementations used the full data set with d = 43 lipid markers. Subsequently, a reduced eight-marker set was obtained using selection. The two columns at the right show the classification performances of a random forest and the eight-item serum lipidomics based classifier respectively biomarker with the selected features. Parameter values were obtained during 1,000 runs using Bootstrap resampling from the test data set. The non-parametric confidence intervals spanning the 2.5^th^ to the 97.5^th^ percentiles of 1,000 Bootstrap resampling runs are given in parentheses.

	k-nearest neighbors	Adaptive boosting	Random forests, complete feature set	Random forests, reduced feature set	Symbolic classifier (Table 3)
Sensitivity, recall [%]	100 (96.91–100)	100 (96.94–100)	100 (96.97–100)	99 (96.84–100)	100
Specificity [%]	85.71 (66.67–100)	97.06 (87.88–100)	100	97.22 (89.65–100)	93.06 (87.5–97.89)
Positive predictive value, precision [%]	95.28 (88.89–100)	99 (96–100)	100	99 96.81–100)	83.33 (71.1–94.12)
Negative predictive value [%]	100 (89.66–100)	100 (91.18–100)	100 (91.67–100)	97.14 (90–100)	100
Balanced accuracy [%]	92.51 (83.33–98.96)	98.39 (93.55–100)	100 (98.48–100)	98.21 (94.34–100)	96.53 (93.75–98.95)
Area under the ROC curve [%]	92.51 (83.33–98.96)	98.39 (93.55–100)	100 (98.48–100)	98.21 (94.35–100)	96.53 (93.75–98.95)
Area under the precision-recall curve [%]	98.87 (93.66–99.05)	98.52 (93.98–99.06)	98.87 (95.23–99.06)	68.89 (55.53–80.15)	68.68 (59.28–77.2)

**Figure 4:**
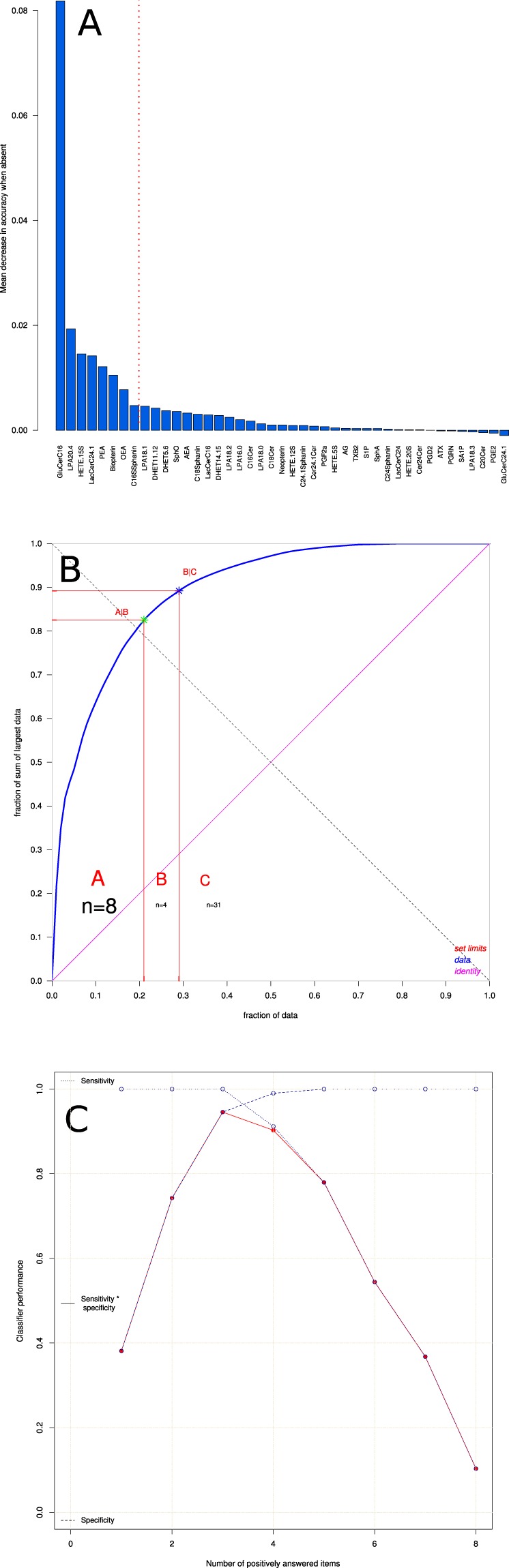
Feature selection and classifier performance. (**A**) Feature selection using random forest machine learning followed by computed ABC analysis of numerical measures of feature importance for classification. A first step of feature selection was analyzing the mean decrease of accuracy over all cross-validated predictions, i.e., the change in the number of observations that were incorrectly classified, when the respective variable was removed. The bar plot shows the decrease in accuracy, as positive values, in descending numerical order. The plot depicts one example out of 1,000 runs and therefore, the order differs from the overall importance order of the parameters as given in Table 3. (**B**) Subsequently, the mean decrease of accuracy associated with each variable was submitted to computed ABC analysis, which is an item selection procedure aiming at identification of most profitable items from a larger list of items. The ABC plot (blue line) shows the cumulative distribution function of the mean decreases in accuracy, along with the identity distribution, *x*_*i*_ = constant (magenta line, i.e., each feature contributes similarly to the classification accuracy (for further details about computed ABC analysis, see^73^). The plot shows results of the same example run as displayed in panel A. (**C**) Plot of the classifier building procedure calculating test performance parameters for every possible sum of positive responses to the rules in Table 3. The solid line shows the product of sensitivity and specificity for different item sums. The dashed lines show the respective test sensitivity and specificity for every sum of positive items. The classification rule tested was “if the sum of the rules that applied was smaller than the actual iteration number then the patient belongs to the “healthy subject” group, else to the “multiple sclerosis” group. The analysis was done iteratively with item sums increasing by a value of one between each iteration. The figure has been created using the R software package (version 3.4.2 for Linux; http://CRAN.R-project.org/)^43^. In particular, the computed ABC analysis was performed and plotted using our R package “ABCanalysis” (http://cran.r-project.org/package=ABCanalysis)^73^.

A computed ABC analysis was applied to the mean decreases in classification accuracy when a particular feature was excluded from random forest building, as calculated during the feature selection step (Fig. 4A). During nested cross-validation including random forests, an out-of-bag error of 2% (range (0–9%) was observed. ABC analysis assigned 2, 3, 4, 5, 6, 7, 8, 9, or 10 items 2, 1, 1, 12, 26, 129, 549, 271 and 9 times to set “A”, respectively. The most frequent size |{ABC set A}| = 8 lipid markers, i.e., the most profitable items for classifier building from the set of candidate features (Fig. 4B), was chosen for further analysis. The eight lipid markers that were most frequently members of the ABC set “A” comprised GluCerC16, HETE15S, LPA20:4, biopterin, OEA, LacCerC24:1, PEA and C16Sphinganin, which belonged to set “A” 1000, 1000, 997, 867, 795, 791, 750, and 582 times, respectively. They were therefore, chosen as components of the final symbolic classifier. The next most frequent lipid marker, HETE12S, was observed 553 times in set “A”, the next, AEA, only 269 times, whereas 24 markers were never chosen as most profitable items. The selected features did not interfere with lipid markers for which evidence of their regulatory functions in association with differential diagnoses of MS (see methods section) was found and therefore, could be verified by experts.

For the item sum based classifier, all possible rule values were iteratively assessed with respect to test performance measures, in particular to the sensitivity in relation to specificity product. The best area under the sensitivity versus specificity curve (Fig. 4C) was provided when a subject was assigned to the healthy subjects group at a sum of <3 positively answered items (Table 3) or to the multiple sclerosis patients group. Most of these rules consisted of an assignment to the multiple sclerosis group when the concentration of the respective lipid mediator fell below a threshold. In other words, most serum lipid mediator concentrations were reduced in multiple sclerosis. Exceptions from this pattern were the ceramide LacCerC24:1 and the sphingolipid C16Sphinganin, which were found at comparatively higher concentrations in the multiple sclerosis patients. Finally, sub-symbolic classifiers based on random forests were created using the reduced d = 8 feature set and the complete d = 43 set of lipid markers. For all classifiers, i.e. (i) the reduced set item sum based classifier, (ii) the reduced set random forest based classifier, and (iii) the complete set random forest based classifier, the classification performance was comparably high (Table 2) with a balanced classification accuracy for association with the multiple sclerosis group of approximately 95% or better.

**Table 3:** Conditions of the prediction of multiple sclerosis. A patient is likely to have multiple sclerosis if at least three of the items (rules) apply, i.e., the conditions given in the rows of the table are true.

Lipid mediator	Threshold*
GluCerC16	<117.93 ng/ml
HETE15S	<0.44 ng/ml
LPA20:4	<71.35 ng/ml
Biopterin	<1.51 ng/ml
OEA	<1.57 ng/ml
LacCerC24:1	>10,880.38 ng/ml
PEA	<1.67 ng/ml
C16Sphinganin	>107.02 ng/ml

^*^Lipid markers had been corrected for age and sex. Therefore, application of above rules to concentration measurements requires a correction as ${Corrected}\,{Value}={Original}\,{Value}+({Ag}{{e}}_{{Subject}}-18)\,\cdot$ $${Slop}{{e}}_{{Age}}-\{\begin{array}{c}if\,male:{\rm{Median}}\,{\rm{Sex}}\,{\rm{Difference}}\,\\ if\,female:0\end{array}\}$$. The value of 18 corresponds to the minimum age in the present cohort and the further parameters of the are given in the following: GluCerC16: Slope_Age_ = 1.6263, Median Sex Difference = −10.2439, HETE15S: Slope_Age_ = 0.0749, Median Sex Difference = 0.3065, LPA20:4: Slope_Age_ = −0.5206, biopterin: Slope_Age_ = 0.0184, Median Sex Difference = 0.4211, OEA: Slope_Age_ = −0.0063, Median Sex Difference = 0.32, LacCerC24:1: Slope_Age_ = −19.0459, Median Sex Difference = −329.1148, PEA: Slope_Age_ = 0.0136, Median Sex Difference = 0.1177, MedianSexDifference = −9.8383, C16Sphinganin: Slope_Age_ = −1.406441, Median Sex Difference = −0.1384429.

## Discussion

The treatment of MS patients should be started as soon as possible after diagnosis has been confirmed in order to reduce residual impairment^3^. In early stages, MS is sometimes difficult to differentiate from other neurologic diseases that mimic MS symptoms. Therefore, there is still a need for a biomarker for the diagnosis of MS^81^. In addition, in the MS context, biomarkers are also being sought for the prediction of the disease course and for monitoring the response to therapy^82,83^. In particular, it would be highly desirable to monitor disease activity during clinically asymptomatic intervals to escalate therapy if needed. Currently, no blood-derived biomarker for MS is available, although many have been suggested^84,85^. In the analysis presented here, supervised machine-learning was successful in detecting the presence or absence of MS at a high accuracy of 96%, with accordingly high scales of sensitivity and specificity. The symbolic classifier, i.e., that most accessible to domain expert interpretation (Table 3), was slightly outperformed by the subsymbolic classifiers, of which that using the whole set of d = 43 markers provided, as expected, the maximum performance measure values (Table 2). The lower accuracy of the symbolic classifier might partially be the effect of somewhat imbalanced case set sizes. Therefore, sensitivity, specificity and balanced accuracy are reported for each classifier type (Table 2), since it is known that accuracy alone might be misleading in cases where the numbers of cases in the positive (n = 102 MS patients) and negative sets (n = 301 controls) are different, i.e. if the sets are imbalanced^86^.

The identified lipid-mediator derived serum biomarkers are biologically plausible and agree with increasing evidence for lipidomic dysregulation of neuro-inflammatory processes and related CNS diseases. Specifically, Cer16 and Cer24:1, the precursors of GluCerC16 and LacCerC24:1, respectively, have been shown to be upregulated in white blood cells isolated from MS patients^21,22^. Moreover, Cer16 and Cer24, being components of extracellular vesicles, might amplify cytokine-induced cell death of myelin-producing oligodendrocytes^87^. Furthermore, HETE15S was shown to be regulated in the cerebrospinal fluid of MS patients^20^. Similarly, enhanced activity of autotaxin, which is an enzyme involved in the biosynthesis of lysophosphatidic acids, was observed in serum samples of MS patients^35^. Biological plausibility of the presently identified components of a lipidomics-based MS biomarker extends to endocannabinoids of which, for example, PEA and OEA have been found in relapse-remitting MS and secondary progressive MS patients^88^. Moreover, experimental data^89^ and a therapeutic benefit of Sativex® which contains cannabidiol and tetrahydrocannabinol, further support a functional relevance of endocannabinoids in MS. Finally, neopterin is an activation marker of the innate immune system with increased levels in autoimmune diseases including the CSF of MS patients^90^.

With a diagnostic accuracy of 95% or better, the present serum lipidomics-based diagnostic biomarker compares to, or outperforms the previously best alternative approaches. For example, based on oligoclonal bands analyzed in paired CSF and serum specimens, MS could be diagnosed at 84.9% sensitivity and 78.9% specificity^91^. Based on gene expression signatures in peripheral blood mononuclear cells, a combination of expression levels of five genes segregated a multiple sclerosis cohort from the respective control cohort with a sensitivity of 91% and specificity of 98%^92^. A miRNA-based marker comprising miR-181c and miR-633 in CSF could differentiate relapsing-remitting from secondary progressive MS courses with 82% specificity and 69% sensitivity^93^. The latter study points towards subtype differences, which were not addressed in the present cohort.

Almost all patients had relapsing remitting MS (RRMS), 55 of 102 with a stable, symptom-free disease under medication with first or second line disease modifying drugs including beta-interferon, fingolimod, natalizumab, fumaric acid or glatiramer acetate, 22 with acute relapse, 6 of them with high-dose prednisolone, and 20 presented with the first course of the disease. Medication may be a confounding factor affecting bioactive lipids. For example, glucocorticoids may reduce LPAs by inhibiting phospholipase A2^94^ and autotaxin^95^, the major producers of extracellular LPAs. Fingolimod may reduce prostaglandins^89^ because it inhibits phospholipase A2^96^ and suppresses the expression or upregulation of cyclooxygenase-2^97^. In addition, fingolimod inhibits autotaxin^34,98^, the latter possibly also affected by natalizumab which interferes with the attachment of autotaxin to the cell surface via integrin. Fingolimod, natalizumab and fumaric acid are all second line drugs for escalation therapy. Therefore, there is a possibility that the observed differences in lipid levels might reflect drug effects. However, at least for the actual medication, this seems unlikely. Specifically, 39 MS patients were receiving no actual medication (Table 1). All were correctly assigned to the MS group by the lipid-based classifier, which is incompatible with a causal role of the varying medication in the shift in serum lipid markers. Similarly, there was no difference in the number of falsely classified patients among the MS subgroups as indicated by non-significant χ^2^ tests.

The present analysis employed random forests followed by ABC analysis as feature selection procedure^71^. The intention was to obtain a symbolic classifier, i.e., a biomarker that is accessible to medical expert explanation. The selection of d = 8 lipid markers out of 43 candidates corresponds to the Miller optimum of d = 7 ± 2 items^99^ that has been proposed to best suit human comprehension^100^. However, it should be noted that the most pronounced effect was observed with GluCerC16 (Fig. 4A). This marker alone provided a high classification performance of more than 90% accuracy that supports a particularly important role of ceramides in the lipid-based pathophysiology of MS^21,22^. Therefore, it is advised to carefully contemplate the diagnosis of MS in cases where GluCerC16 is below the identified concentration threshold (Table 3). The inclusion of d = 8 markers provided further improvement of the classifier performance and made the biomarker more robust than relying on a single laboratory value. Given this observation, and considering that the random forest classifier using all candidate lipid markers did not provide substantially better diagnostic performance than the sum-based reduced feature set classifier. The classifier used outperformed alternative methods of feature selection such as multi-objective parameter tuning^101^ which were not applied, because any possible change in the selection of the less important lipid markers could have provided only a very small improvement in the classifier performance. Moreover, the use of random forests as a basis for the subsequent operations was based on its better performance as compared to alternatives, including kNN and adaptive boosting. Therefore, more alternative classifiers were not assessed in line with the focus of this report on a suitable lipid-mediator concentration based MS biomarker.

Biomarker creation was preceded by an analysis of the data structures, based on the hypothesis that structures coinciding with the prior classification into MS patients and controls provides strong indications that the data set contains information relevant for the diagnosis. This would make it a suitable basis for a supervised machine-learned classifier. In other words, a cluster structure was sought that agreed with the diagnostic groups. As a recent report advised caution against classical clustering methods^48^, different unsupervised machine-learned methods were used, though, Ward clustering was also applied. All methods detected data clusters that almost perfectly agreed with the clinical pre-classification, including the classical Ward clustering that did not show problems occasionally occurring with this method^48^ with the present data. Thus, independent methods supported a data structure in the lipid maker matrix that was compatible with the diagnostic groups.

**Figure 5:**
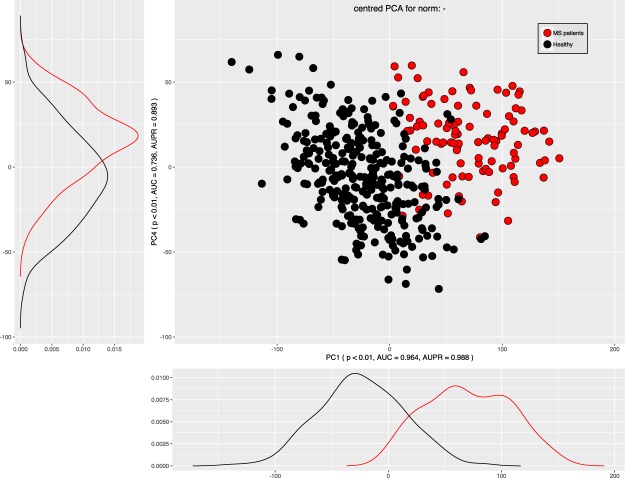
Data structure found in the input space of d = 43 lipid marker serum concentration acquired from patients with multiple sclerosis and from healthy controls. The data structure has been obtained by means of data projection principal component analysis on the non-normalized data as suggested by the results of the PC-corr analysis^102^. The PCA plot associated to this analysis shows the sample separation in the first and fourth component (PC1 versus PC4); the fourth component was chosen as it yielded the best explained variance for unnormalized, centered PCA after the first one. The figure has been created using the R software package (version 3.4.2 for Linux; http://CRAN.R-project.org/)^43^, an R script provided with the description of the PC-corr analysis (pccorrv2.R, https://github.com/biomedical-cybernetics/PC-corr_net)^102^.

To further assess the suitability of the chosen analytical design and to explore differential patterns in the dataset, a PC-corr analysis^102^ was run on the data. Specifically, the PC-corr analysis^102^ provides an algorithm that associates any PCA segregation with a discriminative network of features. Such a network can be inspected for functional modules useful in the definition of combinatorial and multiscale biomarkers from multifaceted omic data. The PC-corr algorithm itself permits to find the best results of a principal component analysis (PCA). PC-corr is an algorithm which is supplementary to PCA. It was developed by Ciucci *et al*.^102^ in 2017 to retrieve the features’ correlations that generate the segregation of the cohorts along a principal component (PC), therefore it is an algorithm that associates to each PC (for which emerges a significant sample discrimination) a discriminative correlation network of the features. As it calculates various quality measures for every combination of PC, normalization and centering, it allows the optimal selection of PC for projection. PCA^103^ uses a rotation of the data, to project the data to a subspace of so-called principal components. The first principal component has the largest possible variance in the data. Each succeeding orthogonal component is chosen for the highest possible remaining variance. PC-corr uses various transformations of the data in the analyses. If its results consist of non-significant separations, as judged by quantitative evaluations expressed as p-value, AUC and AUPR) using any types of normalization and dimension, then a nonlinear dimension reduction is necessary because the data are difficult to linearize by means of different types of normalizations. If the significant separations are found to correspond to particular types of normalization and in dimensions that are not within the first three dimensions of embedding, then the data present nonlinearities that can be addressed by normalization of the data. This analysis was performed using an R script provided with the description of the PC-corr analysis (pccorrv2.R, https://github.com/biomedical-cybernetics/PC-corr_net)^102^. Applying the PC-corr algorithm suggested that a centered PCA, without need of any normalization, produced already a significant segregation of the two cohorts along the first dimension (PC1). Indeed, as reported in the Supplementary Table (line 11), the sample segregation along PC1 had p-value < 0.001, AUC-ROC of 0.96, AUC-PR of 0.99 and explained 25% of the variance (Fig. 5). This result is also confirmed using nonlinear dimension reduction obtained by ESOM and MCE. Hence, the results of nonlinear dimension reduction technique confirm the results obtained by PCA analysis, i.e., the use of non-linear projection methods in the unsupervised analyses of the data structure supported this observations. The chosen analyses (e.g. ESOM and Swarm based projections) also accommodated the intention to favor better performance in embedding by adopting the most advanced nonlinear dimension reduction techniques.

To address possible overfitting, the present data set was split into training and test data sets, nested cross-validation and random resampling being used. Ideally, an independently acquired second data set would have served this purpose. The present analysis provided a basis for further pursuing the development of a serum-lipidomics based MS biomarker. However, instead of acquiring similar data set to again verify the present results, the present results indicate that the next research efforts should rather focus on a marker for early diagnosis. Therefore, a prospective study enrolling patients with unclear symptoms or mono-symptomatic disease typical for early MS stages^2^ is the preferred next step in this project. A further limitation should also be prospectively addressed, namely, the age difference observed here between the MS patients and healthy controls. While this was eliminated in the preprocessing of the present data set, a prospective study should use age-matching in the inclusion criteria. This would eliminate the necessity to rescale the marker concentrations, as shown in the legend of Table 3. Moreover, the present study enrolment neglected the use of contraceptives, recently shown to alter lipid patterns^104^. However, if this had been an important confounder, a separate group would have emerged among the healthy subjects on the U-matrix (Fig. 3), which was not observed.

## Conclusions

Presently, the diagnosis of MS is based on clinical parameters such as the number of relapses, the number and size of the lesions detected by MRI, spinal fluid diagnostics and clinical symptoms characterized by the expanded disability status scale (EDSS). Several clinical criteria are necessary to diagnose MS and to initiate therapy that should be started after the first appearance of clinical symptoms to reduce residual impairment^3^. Therefore, there is a need for a non-invasive biomarker for diagnosis of MS and differentiation of other neurologic diseases, which may mimic MS. In the context of MS, biomarkers are also being sought for the prediction of the disease course and to monitor the response to therapy^82,83^. Using a data-driven approach in a cohort of 102 MS patients and 301 healthy subjects, we have identified a set of serum-based lipid markers of several classes, reported to be modulated in MS (ceramides, sphingolipids, LPAs, endocannabinoids, prostaglandins, pterins, DHETs and HETEs). These could be employed for the diagnosis of MS. Applying unsupervised machine-learning techniques, a data structure, largely agreeing with the clinical diagnoses, was observed that supports the proposal that the set of lipid markers contained information suitable to create a diagnostic tool for multiple sclerosis. Using subsequently supervised machine-learning techniques, a classifier was developed that finally takes the form of a questionnaire with a small set of “yes/no” decisions about lipid biomarker concentrations. The classifier (biomarker) included biologically plausible features, with respect to the identified subset of lipid markers, and predicted MS with an accuracy of approximately 95% in the present data set. This encourages further efforts to establish an MS biomarker based on serum lipidomics^23,55^.

## Electronic supplementary material

Supplementary Dataset 1
